# *Campylobacter coli* Outbreak in Men Who Have Sex with Men, Quebec, Canada, 2010–2011 

**DOI:** 10.3201/eid1905.121344

**Published:** 2013-05

**Authors:** Christiane Gaudreau, Melissa Helferty, Jean-Loup Sylvestre, Robert Allard, Pierre A. Pilon, Michel Poisson, Sadjia Bekal

**Affiliations:** Centre Hospitalier de l’Université de Montréal–Hôpital Saint-Luc, Montreal, Quebec, Canada (C. Gaudreau);; Université de Montréal, Montreal (C. Caudreau, M. Poisson, P.A. Pilon);; Agence de la Santé et des Services Sociaux–Santé Publique de Montréal, Montreal (M. Helferty, J.-L. Sylvestre, R. Allard, P.A. Pilon);; McGill University, Montreal (R. Allard);; Centre Hospitalier de l’Université de Montréal–Hôtel-Dieu, Montreal (M. Poisson);; Laboratoire de Santé Publique du Québec/Institut National de Santé Publique du Québec, Sainte-Anne-de-Bellevue, Quebec, Canada (S. Bekal);; Public Health Agency of Canada, Ottawa, Ontario, Canada (M. Helferty)

**Keywords:** *Campylobacter coli*, emergence, antimicrobial resistance, men, Quebec, Canada, homosexuality, men who have sex with men, bacteria, enteric infections

## Abstract

During September 2010–November 2011, a cluster of erythromycin-susceptible, tetracycline- and ciprofloxacin-resistant *Campylobacter coli* pulsovar 1 infections was documented, involving 10 case-patients, in Montreal, Quebec, Canada. The findings suggested sexual transmission of an enteric infection among men who have sex with men.

*Campylobacter coli* is the second most common species that causes human *Campylobacter* infections (*1*–*3*). Few studies have characterized the differences between the epidemiology and the disease of *C. coli* infections in comparison to *C. jejuni* subsp. *jejuni* infections (*1*–*3*). However, many studies have reported a higher macrolide resistance in *C. coli* than in *C. jejuni* (*1*–*3*). Few *C. coli* outbreaks have been reported to date (*4*,*5*).

## The Study

A retrospective analysis, including the period from January 1, 2010 through December 31, 2011, identified 43 laboratory-confirmed cases of *C. coli* infections reported to the Montreal Public Health Department; among them, 40 cases with antimicrobial drug susceptibility results were further analyzed. Telephone interviews with the case-patients were conducted by using a standardized questionnaire pertaining to symptomatology of the illness, treatment, exposures, sexual orientation (including practices), and HIV status. The questionnaire was mailed to persons who could not be contacted by phone. Hospital charts for 9 or the 10 outbreak case-patients were reviewed retrospectively.

Statistical analyses, using Fisher exact test to calculate the possibilities, were conducted to test for differences in characteristics between case-patients infected with the outbreak etiologic agent, *C. coli* pulsovar 1, and those infected with nonoutbreak *C. coli.* In estimating the odds ratio from a 2 × 2 table that included a zero cell, 0.5 was added to the count in each cell. CIs were calculated by using Miettinen’s test-based method. Statistical analyses were conducted using SPSS software (http://www-01.ibm.com/software/analytics/spss/products/statistics/).

Phenotypic identification of *Campylobacter* isolates at the genus and species levels was confirmed by *cpn60* gene sequencing at Laboratoire de Santé Publique du Québec. *C. coli* strains were identified by direct sequencing of PCR-amplified partial *cpn*60 sequences as described by Hill et al. (*6*). DNA sequences were determined with an ABI 3100 sequencer using a BigDye sequencing kit (Applied Biosystems, Foster City, CA, USA). The sequences were subjected to a BLAST analysis and aligned with the ClustalW program. Phylogenetic analysis was performed using the Lasergene software V6.1 (DNAstar, Madison, WI, USA).

Genetic relatedness was investigated by using pulsed-field gel electrophoresis with *Sma*I according to PulseNet Canada procedures. *Salmonella enterica* serotype Braenderup strain H9812 was used as the marker size in each gel (*7*). For analysis, band position tolerance and optimization values of 1% were used. Similarity coefficient was obtained with the unweighted pair-group method with arithmetic averages. For strains exhibiting similar patterns with *Sma*I, a second enzyme (*Kpn*I) was used to confirm their pulsed-field gel electrophoresis pattern similarity. The PulseNet Canada *Sma*I and *Kpn*I pattern designations for the *C. coli* pulsovar 1 isolate are CASAI.0160 and CAKNI.0078, respectively.

Antimicrobial drug susceptibility testing was determined by using the disk diffusion method for erythromycin, tetracycline, and ciprofloxacin (*8*) and the Etest (AB Biodisk, Solna, Sweden) method for all 12 agents tested (*3*). β-lactamase susceptibility was determined as reported (*9*).

**Figure 1:**
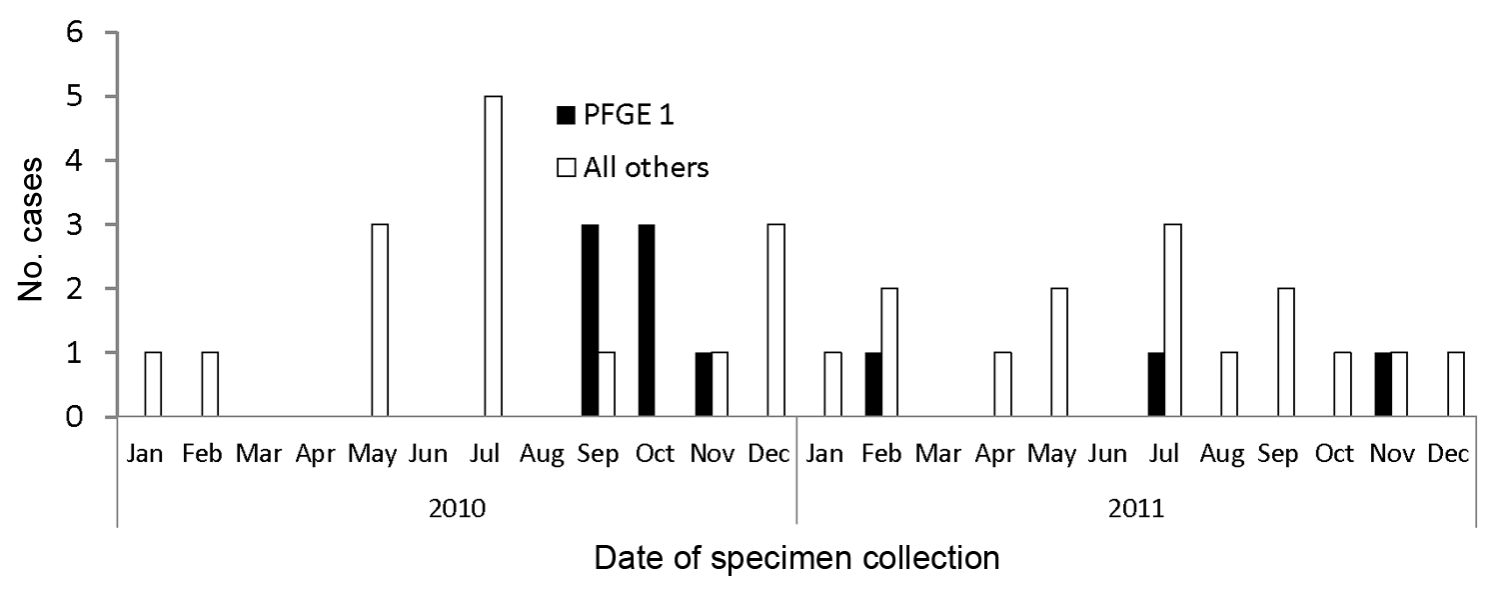
Number of cases of *Campylobacter coli* infection reported to Montreal Public Health, Quebec, Canada, 2010–2011. PFGE, pulsed-field gel electrophoresis.

**Figure 2:**
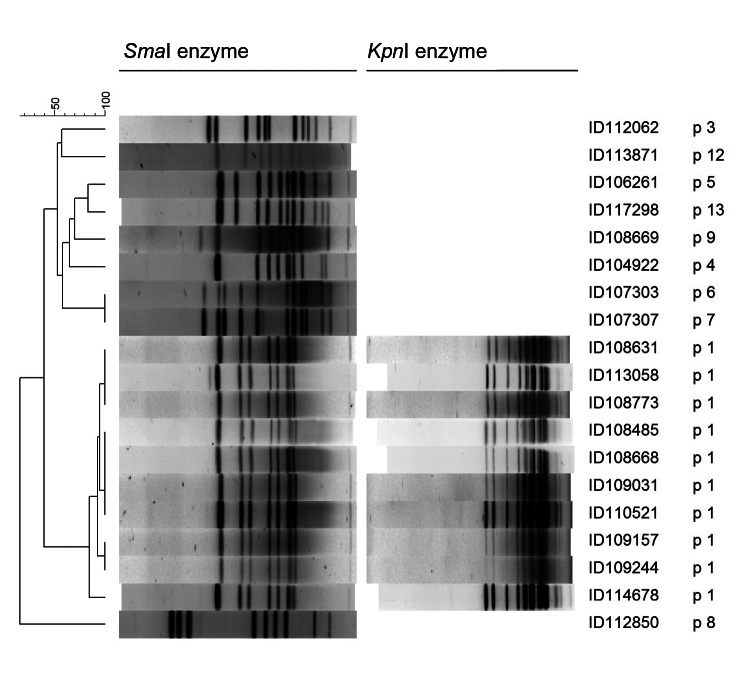
Pulsed-field gel electrophoresis (PFGE) patterns of erythromycin-susceptible, tetracycline- and ciprofloxacin-resistant, *Campylobacter coli* for *Sma*I (19 isolates) and *Kpn*I (10 isolates) enzymes, Montreal, Quebec, Canada, 2010–2011. Scale bar indicates percent similarity. p, pulsovar.

From September 2010 through November 2011, in Montreal, 10 men, 26–57 years of age, were found to be infected with an erythromycin-susceptible, tetracycline- and ciprofloxacin-resistant *C. coli* pulsovar 1; these men were defined as the outbreak-associated case-patients (Figure 1). An additional 5 women and 4 men were infected with an erythromycin-susceptible, tetracycline- and ciprofloxacin-resistant *C. coli* strain; however, 9 different pulsovars were involved (Figure 2). Microbiology laboratories at Centre Hospitalier de l’Université de Montréal documented 9 of the 10 outbreak cases, but did not isolate erythromycin-susceptible, tetracycline- and ciprofloxacin-resistant *C. coli* from December 2011 through November 2012.

Compared with the 30 nonoutbreak case-patients for whom suceptibility results were available, the 10 outbreak case-patients were more likely to be male (p = 0.010), to be 20–59 years of age (p = 0.010), to be men who have sex with men (MSM) (p = 0.0001), to be HIV positive (p = 0.001), and to have had sexual relations within 2 weeks of the beginning of gastrointestinal symptoms (p = 0.017) (Table 1). Of the 8 HIV-positive patients, the CD4 cell count was 210 × 10^6^/L for 1 man and 440–1,150 × 10^6^/L for the 6 other patients, and the HIV viral load was 68 copies/mL for 1 patient and <40 copies/mL for the 5 other patients for whom these data were known.

**Table 1:** Statistically significant differences between case-patients with outbreak-related *Campylobacter coli* pulsovar 1 (n = 10) and nonoutbreak *C. coli* (n = 30) infections, Montreal, Quebec, Canada, 2010–2011*

Characteristic	No. outbreak case-patients	No. nonoutbreak case-patients	OR (95% CI)	p value
Male sex, n = 40	10/10	17/30	16.20 (1.88–139.00)	0.010
Age 20– 59 y, n = 40	10/10	17/30	16.20 (1.88–139.00)	0.010
Had sexual relations within the incubation period, n = 23	6/6	7/17	18.20 (1.87–177.00)	0.017
MSM, n = 17	9/10	0/7	95.00 (8.29–1,089.00)	0.0001
HIV positive, n = 15	8/9	0/6	73.67 (6.09–891.00)	0.001

*OR, odds ratio; MSM, men who have sex with men.

Seven men (70%), all MSM, lived in surrounding neighborhoods of Montreal’s so-called Gay Village. The antimicrobial drug treatment regimen was known for 1 outbreak case-patient who received oral azithromycin. In the previous 15 years, 1 HIV-positive man (who also had sex with men) and had *C. coli* pulsovar 1 and *S. flexneri* in fecal specimens, exhibited 7 other sexually transmitted diseases. Among the 10 outbreak case-patients, 2 HIV-positive MSM were simultaneously infected with an *S. flexneri* isolate, and 1 of these 2 patients experienced *C. coli* septicemia.

The following data were reported for 10 outbreak *C. coli* and 30 nonoutbreak *C. coli* case-patients, respectively: diarrhea, 100% (6/6) and 88% (23/26); abdominal cramps, 60% (3/5) and 83% (19/23); blood in stool specimen, 20% (1/5) and 30% (6/20); fever, 60% (3/5) and 61% (14/23); and hospitalization, 17% (1/6) and 19% (5/26) (p>0.05 for all data). Exposures to potential sources of infection did not differ between outbreak and nonoutbreak case-patients. All patients reported having consumed meat, dairy products, tap water or commercially bottled only, and no nonchlorinated water. Exposures to animals, farms, and other persons with known cases of diarrhea were rarely reported. Travel history outside of the island of Montreal in the 2 weeks before symptom onset was documented in none (0/6) of *C. coli* pulsovar 1 case-patients and in 48% (12/25) of other *C. coli* case-patients (p = 0.059); all 12 had traveled outside Canada.

The 10 outbreak isolates were susceptible to erythromycin, azithromycin, ampicillin, gentamicin, imipenem, clindamycin, chloramphenicol, and tigecycline and were β-lactamase negative. All 10 were resistant to ciprofloxacin, nalidixic acid, tetracycline, and cefotaxime (Table 2).

**Table 2:** Antimicrobial drug susceptibility results for *Campylobacter coli* pulsovar 1 isolates from 10 patients, Montreal, Quebec, Canada, 2010–2011*

Antimicrobial agent†	MIC (mg/L)	Interpretation
Erythromycin	2–4	S
Azithromycin	0.25–0.5	S
Tetracycline	128–256	R
Ciprofloxacin	>32	R
Nalidixic acid	>256	R
Ampicillin	2–4	S
Gentamicin	0.5–1	S
Cefotaxime	>32	R
Imipenem	0.06–0.12	S
Clindamycin	0.25–0.5	S
Chloramphenicol	2–4	S
Tigecycline	≤0.015	NA
β-lactamase‡	Negative	–

*S, susceptible; R, resistant; NA, not available; –, not applicable. †The susceptibility and resistance breakpoints were Clinical and Laboratory Standards Institute (CLSI) *Campylobacter* breakpoints for erythromycin, tetracycline and ciprofloxacin (*10*), National Antimicrobial Resistance Monitoring System *Campylobacter* breakpoints for azithromycin and clindamycin (*11*), no breakpoints available for tigecycline and CLSI *Enterobacteriaceae* breakpoints for the 6 other antimicrobial agents (*12*). ‡β-lactamase susceptibility was determined as described (*9*).

## Conclusions

Epidemiologic and molecular data confirmed a cluster of erythromycin-susceptible, tetracycline- and ciprofloxacin-resistant, *C. coli* pulsovar 1 infections in MSM in Montreal, Quebec, Canada, during September 2010–November 2011. The epidemiologic data reported in Table 1, the 14-month outbreak duration, the simultaneous *S. flexneri* infection in 2 HIV-positive MSM, and the absence of any reported common food exposure suggest a sexually transmitted enteric infection. A cluster of erythromycin- and ciprofloxacin-resistant, tetracycline-susceptible *C. jejuni* subsp. *jejuni* infections from 1999 through 2001 (*13*) and 7 clusters of *Shigella* spp. infections from 1999 through 2011 (*14**,**15**;* unpub. data), which were sexually transmitted, have been documented in MSM in Montreal and surrounding neighborhoods. Among MSM, *Shigella* spp. infection is, in most cases, sexually transmitted (*15*).

*C. coli* infection clusters are infrequently reported (*4*,*5*). *Campylobacter* should be identified to species level by phenotypic and, if needed, by molecular characterization. Association of cluster cases with the correct *Campylobacter* species is the first step of suspecting an outbreak and can lead to improved outbreak detection. Antimicrobial drug susceptibility testing, at least to erythromycin and ciprofloxacin, is recommended for every isolate (*10*). The erythromycin, ciprofloxacin, and tetracycline susceptibilities were epidemiologic markers in the *Campylobacter* spp. clusters documented in Montreal (present study; *13*). Nine different pulsovars were documented in 9 nonoutbreak case-patients, indicating a high heterogeneity of *C. coli*.

If necessary, the first-choice antimicrobial drug treatment for patients infected with *C. coli* pulsovar 1 would be a macrolide as it is for *C. jejuni* and *C. coli* enteric infections because of increasing fluoroquinolone resistance in these bacteria (*1*–*3*). HIV-positive or AIDS patients may have a higher incidence of *Campylobacter* infections with more septicemia and more complicated outcome than healthy patients have (*1*,*2*). MSM should be counseled on methods to avoid or reduce the risk of sexual transmission of enteric infections such as those caused by *Campylobacter* or *Shigella* (*13*).

## References

[1] Allos BM, Blaser MJ. *Campylobacter jejuni* and related species. In: Mandell GL, Bennett JE, Dolin R, editors. Principles and practice of infectious diseases, 7th ed. Philadelphia: Elsevier Churchill Livingston; 2010. p. 2793–802.

[2] Blaser MJ, Engberg J. Clinical aspects of *Campylobacter jejuni* and *Campylobacter coli* infections. In: Nachamkin I, Szymanski CM, Blaser MJ, editors. *Campylobacter,* 3rd ed. Washington (DC): American Society for Microbiology; 2008. p. 99–121.

[3] Fitzgerald C, Nachamkin I. *Campylobacter* and *Arcobacter.* In: Versalovic J, Carroll KC, Funke G, Jorgensen JH, Landry ML, Warnock DW, editors. Manual of clinical microbiology, 10th ed. Washington (DC): American Society for Microbiology; 2011. p. 885–99.

[4] Gallay A, De Valk H, Cournot M, Ladeuil B, Hemery C, Castor C, A large multi-pathogen waterbone community outbreak linked to faecal contamination of a groundwater system, France, 2000. Clin Microbiol Infect. 2006;12:561–70. 10.1111/j.1469-0691.2006.01441.x 16700706

[5] Wardak S, Sadkowska-Todys M. The first report on *Campylobacter coli* family outbreak detected in Poland in 2006. Euro Surveill. 2008;13:8052 . 18445405

[6] Hill JE, Paccagnella A, Law K, Melito PL, Woodward DL, Price L, Identification of *Campylobacter* spp. and discrimination from *Helicobacter* and *Arcobacter* spp. by direct sequencing of PCR-amplified *cpn60* sequences and comparison to cpnDB, a chaperonin reference sequence database. J Med Microbiol. 2006;55:393–9. 10.1099/jmm.0.46282-0 16533986

[7] Hunter SB, Vauterin P, Lambert-Fair MA, Van Duyne MS, Kubota K, Graves L, Establishment of a universal size standard strain for use with the PulseNet standardized pulsed-field gel electrophoresis protocols: converting the national databases to the new size standard. J Clin Microbiol. 2005;43:1045–50. 10.1128/JCM.43.3.1045-1050.2005 15750058 PMC1081233

[8] Gaudreau C, Girouard Y, Gilbert H, Gagnon J, Bekal S. Comparison of disk diffusion and agar dilution methods for erythromycin, ciprofloxacin and tetracycline susceptibility testing of *Campylobacter coli* and for tetracycline for *Campylobacter jejuni* subsp. *jejuni.* Antimicrob Agents Chemother. 2008;52:4475–7. 10.1128/AAC.00767-08 18838597 PMC2592876

[9] Lachance N, Gaudreau C, Lamothe F, Turgeon F. Susceptibilities of β-lactamase–positive and –negative strains of *Campylobacter coli* to β-lactam agents. Antimicrob Agents Chemother. 1993;37:1174–6. 10.1128/AAC.37.5.1174 8390812 PMC187926

[10] Clinical and Laboratory Standards Institute. Methods for antimicrobial dilution and disk susceptibility testing for infrequently-isolated or fastidious bacteria: approved guidelines; no. M45–A2, vol. 30, no. 18. Wayne (PA): The Institute; 2010.

[11] Centers for Disease Control and Prevention. 2010. National Antimicrobial Resistance Monitoring System-enteric bacteria (NARMS) 2009 annual report [cited 2011 Jun 29]. http://www.cdc.gov/narms/pdf/NARMSAnnualReport2009_508.pdf.

[12] Clinical and Laboratory Standards Institute. Performance standards for antimicrobial susceptibility testing; 22th informational supplement; no. M100–S22, vol. 32, no. 3. Wayne (PA): The Institute; 2012.

[13] Gaudreau C, Michaud S. Cluster of erythromycin- and ciprofloxacin-resistant *Campylobacter jejuni* subsp. *jejuni* from 1999 to 2001 in men who have sex with men, Québec, Canada. Clin Infect Dis. 2003;37:131–6. 10.1086/375221 12830417

[14] Gaudreau C, Bruneau A, Ismaïl J. Outbreak of *Shigella flexneri* and *Shigella sonnei* enterocolitis in men who have sex with men, Québec, 1999 to 2001. Can Commun Dis Rep. 2005;31:85–90 . 15875326

[15] Gaudreau C, Ratnayake R, Pilon PA, Gagnon S, Roger M, Levesque S. Ciprofloxacin-resistant *Shigella sonnei* among men who have sex with men, Canada, 2010. Emerg Infect Dis. 2011;17:1747–50. 10.3201/eid1709.102034 21888811 PMC3322076

